# Drosophila UBE3A regulates satiety signaling through the Piezo mechanosensitive ion channel

**DOI:** 10.21203/rs.3.rs-3101314/v1

**Published:** 2023-07-03

**Authors:** Benjamin Geier, Logan Neely, Eli Coronado, Lawrence T. Reiter

**Affiliations:** University of Tennessee Health Science Center; University of Tennessee Health Science Center; University of Tennessee Health Science Center; University of Tennessee Health Science Center

## Abstract

Angelman syndrome (AS) is a rare neurogenetic disorder characterized by developmental delays, speech impairments, ataxic movements, and in some cases, hyperphagic feeding behavior. Loss of function mutations, loss of expression from the maternal allele or absence of maternal UBE3A result in AS. Recent studies have established a connection between *UBE3A* and the mechanosensitive ion channel *PIEZO2*, suggesting the potential role of UBE3A in the regulation of PIEZO channels. In this study, we investigated the role of *Drosophila UBE3A* (*Dube3a*) in *Piezo* associated hyperphagic feeding behavior. We developed a novel assay using green fluorescent protein (GFP) expressing yeast to quantify gut distention in flies with *Piezo* and *Dube3a* mutations. We confirmed that *Dube3a*^*15b*^ loss of function flies displayed gut distention to almost identical levels as *Piezo*^*KO*^ flies. Further analysis using deficiency (Df) lines encompassing the *Dube3a* locus provided proof for a role of *Dube3a* in satiety signaling. We also investigated endogenous *Piezo* expression across the fly midgut and tracheal system. Piezo protein could be detected in both neurons and trachea of the midgut. Overexpression of *Dube3a* driven by the *Piezo* promoter resulted in distinct tracheal remodeling within the midgut. These findings suggest that *Dube3a* plays a key role in the regulation of Piezo and that subsequent dysregulation of these ion channels may explain the hyperphagic behavior observed in 32% of cases of AS. Further investigation will be needed to identify the intermediate protein(s) interacting between the Dube3a ubiquitin ligase and Piezo channels, as Piezo does not appear to be a direct ubiquitin substrate for UBE3A in mice and humans.

## Introduction

Angelman syndrome (AS) is a rare neurogenetic disorder with an incidence of 1/15,000 births and characterized by severe developmental delays, speech impairments, ataxic movements, epilepsy, and frequent laughter [1]. AS arises from the loss of expression of a single paternally imprinted copy of the *UBE3A* gene, a HECT domain E3A ubiquitin ligase, also known as E6AP [2, 3]. While the majority of AS cases occur as a result of deletion of the maternal 15q11.2-q13.1 locus [4], loss of function (LoF) mutations that exclusively affect *UBE3A* on the maternal allele will also result in AS [5]. Biochemical studies investigating point mutations in UBE3A reveal that any loss or reduction of catalytic activity plays a critical role in the development of AS [6]. Large maternal deletions comprise 75% of all AS cases, point mutations of maternal *UBE3A* comprise 20% of cases, imprinting center defects make up 3%, and paternal uniparental disomy (pUPD) causes up to 2% of causes [7]. Previous work suggested that, unlike the major deletion class, only pUPD patients display hyperphagic feeding behavior [8, 9]. However, a newer large-scale AS cohort study suggests this hyperphagic phenotype may be present in all AS subtypes [10].

A new connection between UBE3A and the mechanosensitive ion channel PIEZO2 has recently been established. In this study, loss of *Ube3a* in an AS mouse model and human AS cell lines resulted in decreased PIEZO2 activity and protein expression [11]. Piezo proteins are pore-forming subunits of ion channels that activate in response to mechanical stimuli [12]. The human genome encodes two channel isoforms, PIEZO1 and PIEZO2, which are expressed in a myriad of tissues (kidneys, vasculature, Merkel cells, and others) [13–17]. In *D. melanogaster* there is only one copy of the Piezo ion channel gene, but it shares equal homology for both mammalian isoforms [18]. Like mammals, Drosophila express *Piezo* in the crop (stomach) and intestine. Knockout of *Piezo* in Drosophila results in a severe gut distention phenotype, indicative of hyperphagia [19, 20]. The high homology between fly and mammalian Piezo, in addition to the connection between UBE3A and PIEZO2 recently described in *Ube3a* deficient mice, prompted us to look for a connection between *Dube3a* and *Piezo* in flies in order to investigate hyperphagia associated with AS.

Here we investigated the role of Dube3a (the fly UBE3A homolog) in hyperphagic feeding behavior and its role in altering expression of Piezo in cells of the midgut. We established a novel feeding assay incorporating green fluorescent protein (GFP) expressing yeast for quantitative analysis of gut distention. Using this new assay, we show clear connections between *Dube3a*, *Piezo* and hyperphagia. Furthermore, to assess changes in *Piezo* expression within the fly midgut, we generated a new fly that expresses GFP-tagged Piezo and also overexpresses *Dube3a*. By driving expression of our new flies under the *Piezo* promoter, we were able to identify morphological changes in *Piezo* positive terminal tracheal cells (TTC) within the fly midgut. The identification of Dube3a as a Piezo regulator provides a new avenue for potential therapeutics of AS related hyperphagia.

## Results

### Dube3a loss of function flies display gut distention phenotype indicative of hyperphagia

**Figure 1:**
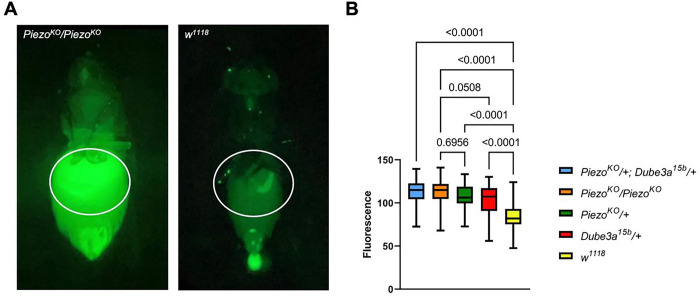
*Dube3a* LoF recapitulates *Piezo*^*KO*^ gut distention phenotype. **A)** Image of *Piezo*^*KO*^ homozygote to *w*^*1118*^ fly after ingesting GFP+ yeast. Region of interest (white outline) for fluorescent normalization and quantification. **B)** Feeding analysis of *Piezo*^*KO*^*/+; Dube3a*^*15b*^*/+* (n=37); *Piezo*^*KO*^*/Piezo*^*KO*^ (n=48), *Piezo*^*KO*^/+ (n=41), *Dube3a*^*15b*^ (n=45), and *w*^*1118*^ (n=51). Significant gut distention phenotypes were observed in all groups when compared to *w*^*1118*^ control flies. *Piezo*^*KO*^*/Piezo*^*KO*^ flies did not show significant distention versus *Dube3a*^*15b*^ and *Piezo*^*KO*^*/+* flies. Analysis by one-way ANOVA p_value_ ≤ 0.05 for significance.

To explore the connection between UBE3A and PIEZO2, we generated a novel feeding assay using GFP expressing *Saccharomyces cerevisiae* (brewer’s yeast). Flies were starved prior to the assay and then fed GFP expressing yeast for fluorescent quantification of gut distention. The GFP signal can be detected in the crop and gut of flies after consumption for accurate quantification. To determine if loss of Dube3a affects feeding behavior, we used a previously published *Dube3a* LoF mutant (*Dube3a*^*15b*^) [21] to compare to flies that have a known gut distention phenotype due to loss of *Piezo* [19]. Homozygous *Piezo*^*KO*^ mutants display significant gut distention, whereas *w*^*1118*^ animals feed at a basal levels and do not show distention of the crop region. Quantitative comparison of abdomen sizes for *Piezo*^*KO*^ mutants to *w*^*1118*^ flies showed a significant difference in fluorescent signal and abdomen size in a region of interest (ROI) surrounding the crop (Fig. 1A). Using *Dube3a*^*15b*^ mutants, we compared feeding behavior among *w*^*1118*^, *Piezo*^*KO*^/*Piezo*^*KO*^, *Piezo*^*KO*^/+, *Dube3a*^*15b*^/+, and *Dube3a*^*15b*^/*Piezo*^*KO*^ animals. Quantitative fluorescent intensities showed significant elevation in gut distention for all genotypes when compared to *w*^*1118*^. Additionally, *Dube3a*^*15b*^ mutants showed elevated gut distention akin to *Piezo*^*KO*^/+ flies. Addition of the *Piezo*^*KO*^ mutation (*Dube3a*^*15b*^/*Piezo*^*KO*^) increased hyperphagic feeding behavior to the same level as the *Piezo*^*KO*^/*Piezo*^*KO*^ flies (Fig. 1B). These findings suggest a possible role of Dube3a in the regulation of satiety signaling through Piezo regulation.

### Deficiencies that uncover the Dube3a locus confirm Dube3a as a regulator of satiety signaling

**Figure 2:**
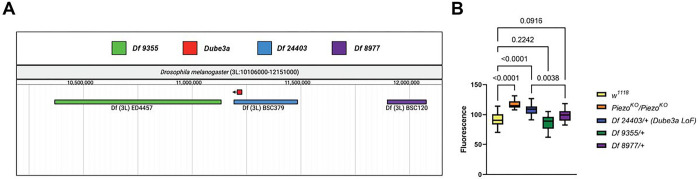
3L deficiency flies containing *Dube3a* LoF recapitulate gut distention phenotype. **A)** Genomic region encompassing *Dube3a* locus on chromosome 3L. Genomic map captured through FlyBase JBrowse feature. **B)** Distention analysis of *w*^*1118*^ (n=29), *Piezo*^*KO*^*/Piezo*^*KO*^ (n=25), *Df 24403/+* (n=23), *Df 9355/+* (n=28), *Df 8977/+* (n=23). *Piezo*^*KO*^*/Piezo*^*KO*^ positive control and deficiency flies that uncover the *Dube3a* locus had significant gut distention phenotypes when compared to control *w*^*1118*^ flies. Df *24403/+* had a significantly increased gut distention phenotype when compared to Df *8977/+.* Analysis by one-way ANOVA p_value_ ≤ 0.05 for significance.

To confirm *Dube3a’s role* in the gut distention phenotype using a different genetic background, we tested several Bloomington Drosophila Stock Center (BDSC) deficiency (Df) lines. The Df lines have small and large deletions across the entirety of the Drosophila genome [22, 23]. To ensure a direct effect of loss of *Dube3a* on satiety signaling, we performed the GFP feeding assay on three Df lines on Drosophila 3L. Two lines, *Df 9355* and *Df 8977* flank the *Dube3a* gene, while *Df 24403* is lacking the *Dube3a* gene (Fig. 2A). To eliminate other background effects, we removed *CyO* and TM6B, *Tb, Hu* balancers by out-crossing all Df lines to *w*^*1118*^ flies prior to testing. Comparison of quantitative gut distention revealed a significant increase in feeding behavior of *Df 24403* (*Dube3a* LoF) but not for flanking Df lines (Fig. 2B). While *Df 8977* showed an elevated fluorescent intensity signal, it was not significantly different from *w*^*1118*^. An unpaired t-test comparing *Df 24403* and *Df 8977* showed a significant increase in distention for *Dube3a* LoF mutants, confirming the relationship between *Dube3a* and hyperphagic feeding behavior (Fig. 2B), regardless of genetic background. These results suggest that loss of UBE3A could cause hyperphagia though down regulation of PIEZO2, parallel to their roles in the ataxia phenotype in the AS mouse model [11].

### Fluorescent imaging of Piezo:GFP flies reveals distinct Piezo patterning across Drosophila midgut

**Figure 3:**
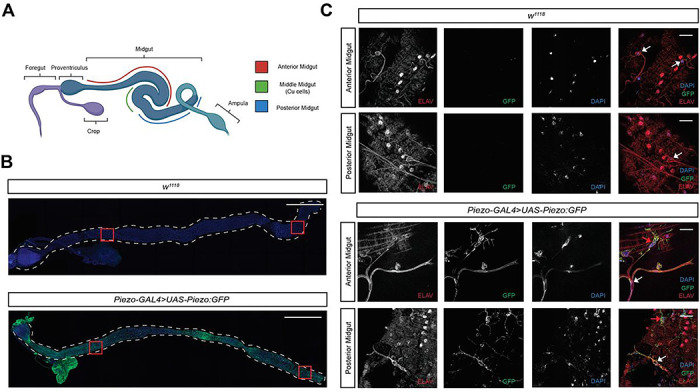
Expression pattern of Piezo protein across Drosophila midgut. **A)** Diagram detailing the anatomical portions of the Drosophila intestinal system (created with BioRender). **B)** Green channel (488nm) images of *w*^*1118*^ midgut show no auto florescent signal. *Piezo>Piezo:GFP* intestinal tract shows detectable GFP signal spanning the entirety of the midgut. **C)** Anterior and posterior midgut IF images of *w*^*1118*^ fly show *elav* positive cells (white arrows) with no detectable GFP signal. *Piezo>Piezo:GFP* IF images displaying elav positive cells (white arrows) overlapping with GFP signal. Red arrows point to TTCs. Whole intestine scale bar, 500μM. Anterior and posterior zoom of midgut scale bar, 20μM.

The gastrointestinal (GI) system of Drosophila is a tubular structure consisting of a foregut, midgut, and hindgut, responsible for the intake, breakdown, and absorption of nutrients (Fig. 3A) [24]. Before assessing the relationship of Dube3a and Piezo in the fly gastrointestinal (GI) system, we determined which cells in the gut normally express Piezo. We performed immunofluorescent (IF) staining on whole Drosophila intestines for both *Piezo > Piezo:GFP* and *w*^*1118*^ flies. Staining of *w*^*1118*^ intestines with antibodies against GFP, neurons (*elav*), and counterstained for nuclei (DAPI) revealed both neuronal and nuclear staining in the anterior and distal midgut but no GFP signal detected (Fig. 3B). Using a Piezo specific GAL4 driver [25] to drive expression of UAS-*Piezo:GFP*, we found that *Piezo > Piezo:GFP* flies showed GFP signal in the proventriculus, crop, and all sections of the midgut. Additionally, neuronal and nuclear staining showed strongly colocalization of a-GFP with a-elav, suggesting specificity for Piezo in gut neurons (Fig. 3C).

### Overexpression of Dube3a results in tracheal remodeling of the anterior midgut

**Figure 4:**
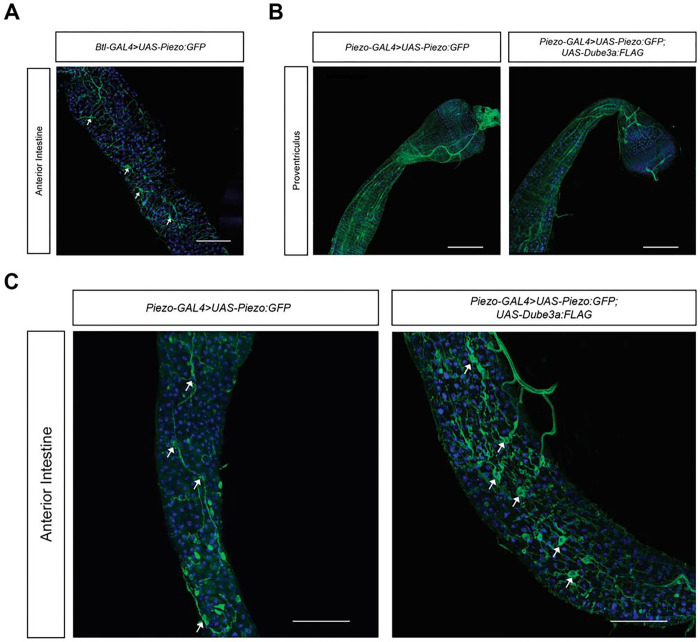
*Dube3a* overexpression drives tracheal remodeling of anterior midgut. **A)** Anterior intestine of *Btl>Piezo:GFP*showing Piezo-positive terminal tracheal cells (white arrows). **B)** Images of proventriculus of *Piezo>Piezo:GFP* and *Piezo>Piezo:GFP; Dube3a:FLAG*. No noticeable changes in *Piezo* positive cells were noted within the proventriculus. **C)** Images of anterior intestine of *Piezo>Piezo:GFP* and *Piezo>Piezo:GFP; Dube3a:FLAG*. Anterior midgut of *Piezo>Piezo:GFP; Dube3a:FLAG* flies display tortuous remodeling of trachea and bloated TTCs. Images taken at 63X. Scale bar, 100μM.

Along with *elav*-positive cells, imaging of *Piezo > Piezo:GFP* flies showed GFP-positive cells with elaborate arborization patterns (red arrows Fig. 3C). These cells displayed a similar morphology to TTCs [26]. To confirm that these are TTC and not neurons, we drove the expression of *Piezo:GFP* using the tracheal GAL4 driver breathless (*btl*-GAL4). Fluorescent imaging of btl-GAL4 > *Piezo:GFP* revealed a similar morphological pattern in the midgut to *Piezo > Piezo:GFP* flies (white arrows Fig. 4A). These results imply that Piezo is also expressed within the tracheal system.

We next investigated how changes in *Dube3a* affect Piezo expression and distribution in the gut as well as gut morphology. We generated a double UAS line UAS-*Piezo:GFP*; UAS-*Dube3a*:*FLAG* to visualize Piezo while also elevating Dube3a levels. This line expresses the Piezo:GFP fusion protein at basal levels in conjunction with the overexpression of Dube3a-FLAG in Piezo expressing cells. Western blot analysis on fly head extracts from *w*^*1118*^ and *glass multimer report* (GMR) *GMR > Piezo:GFP; Dube3a:FLAG* animals confirmed the presence of Dube3a-FLAG in double UAS line (**Fig. S1**). Protein expression studies using a-FLAG antibody revealed a band at 120kDa exclusively in *GMR > Piezo:GFP; Dube3a:FLAG* flies, confirming the presence of Dube3a-FLAG expression in the recombinant flies. It became apparent that *Piezo > Piezo:GFP; Dube3a:FLAG* flies did not emerge at 25°C, so crosses were set at 18°C to reduce the level of Dube3a-FLAG overexpression in the *Piezo > Piezo:GFP; Dube3a:FLAG* offspring. Although there was significant lethality for Piezo > Dube3a-FLAG flies at 18°C, at least two *Piezo > Piezo:GFP; Dube3a:FLAG* flies could be generated for study (**Fig. S2**). Confocal imaging of these two *Piezo > Piezo:GFP; Dube3a:FLAG* flies showed no major changes in the proventriculus but a noticeable change in the TTC morphology within the anterior midgut (Fig. 4B & 4C). *Piezo > Piezo:GFP; Dube3a:FLAG* TTC projections exhibited a tortuous appearance when compared to the *Piezo > Piezo:GFP* counterparts. Additionally, overexpression of Dube3a-FLAG caused bloating of TTC cell bodies when compared to control lines (white arrows Fig. 4C). These results indicate that overexpression of *Dube3a* in normally *Piezo* expressing cells of the gut results in severe tracheal remodeling of the Drosophila midgut, potentially through the dysregulation of Piezo.

## Discussion

Although previously thought to be a feature unique to the pUPD class of AS [8], more recent research has identified hyperphagia as a common symptom across all AS subtypes [10]. Collaborative work from our laboratory also recently established a connection between UBE3A and the mechanosensitive ion channel PIEZO2, leading to the hypothesis that UBE3A might indirectly regulate PIEZO channels, contributing to the both ataxia [11] and hyperphagia in AS. Here we investigated this hypothesis and establish a link between the loss of *Dube3a* and the emergence of hyperphagic feeding behavior in *Dube3a* mutant flies, resembling hyperphagia in *Piezo*^*KO*^ flies and establishing that these two genes may act in the same pathway to control satiety. Additionally, intestinal imaging of *Piezo* driven overexpression of *Dube3a* revealed noticeable tracheal remodeling within the fly midgut further supporting the role of Dube3a in the Drosophila satiety signaling.

We also developed a new assay utilizing GFP expressing yeast to quantify gut distention. Equivalent fluorescent intensities were detected between *Dube3a*^*15b*^ and *Piezo*^*KO*^ flies in this assay, suggesting a putative role for Dube3a in satiety signaling. This connection between satiety and Dube3a was confirmed using Df lines that uncover the Dube3a locus (Fig. 2B). We previously demonstrated that *Dube3a*^*15b*^ mutants have significantly fewer actin filaments than their wild type counterparts [27]. Actin is essential for the trafficking of proteins to the membrane [28]. Studies have shown that a functional cytoskeleton is also required for proper Piezo activity [29, 30]. Here we propose that the dysregulation of actin filaments, via loss of Dube3a, inhibits Piezo trafficking/activity resulting in hyperphagia.

Gut imaging experiments provided insights not only into the normal expression pattern of Piezo within the Drosophila midgut, but also the effects of Dube3a overexpression on normal gut morphology. Piezo expressing neurons were detected in both the anterior and posterior midgut. These Piezo expression patterns were identical to other studies investigating Piezo within the adult midgut but were extended in this study to include co-localization with neuronal and tracheal markers [25]. Notably, *Piezo:GFP* expression under the breathless promoter (*btl*-GAL4) showed a pattern similar to *Piezo > Piezo:GFP* flies for patterning across the midgut, indicating the presence of Piezo channels within the tracheal system.

The Drosophila tracheal system, akin to vasculature in humans, serves as a complex tubular system that provides oxygen to cells [26]. Similarly, mammalian Piezo1 channels are present in vasculature, playing a role in pathfinding and angiogenesis [31–33]. Mice deficient for *Piezo1* also die during mid gestation stage emphasizing the role of Piezo channels in proper development [34]. Consistent with these findings, attempts to analyze *Piezo > Piezo:GFP; Dube3a-FLAG* flies were hindered by low eclosure rates, suggesting an effect on *Piezo* expression and or function after *Dube3a* overexpression.

In *Piezo > Piezo:GFP; Dube3a-FLAG* flies, the midgut displayed a highly disorganized tracheal phenotype. While tracheal branching is not fully understood, trachea increase their arborization under both physical and cellular stress conditions to meet the metabolic demands of surrounding cells [35, 36]. Although the direct cause of tracheal remodeling in these flies is unknown, swelling of tracheal cells implies stressed intestinal conditions due to *Dube3a* overexpression. The Drosophila midgut contains enteroendocrine cells (EC) and enteric neurons, which play roles in satiety signaling [37, 38]. Furthermore, Piezo is expressed in subsets of intestinal stem cells (ISCs), contributing to EC differentiation [25]. The negative effect of Dube3a on Piezo channels may disrupt proper satiety signaling through dysregulation of enteric neurons, ISCs and ECs, but further studies will be needed to narrow down the exact cell types where Dube3a can regulate Piezo in the gut.

In summary, these studies show a clear connection between *Dube3a* loss of function and hyperphagic feeding behavior. Imaging analysis also revealed a tortuous appearance of *Piezo* positive trachea within the fly midgut supporting the role of Dube3a in altering *Piezo* expression and or function during development. Although the intermediate protein(s) involved in regulating Piezo expression remain(s) unidentified, current evidence suggest cofilin as a potential Dube3a polyubiquitination substrate that could affect Piezo function in the membrane [11]. The findings here strengthen the connection between Dube3a and Piezo and their potential role in the regulation of hyperphagia in AS.

## Materials and Methods

### Fly stocks

Fly stocks were maintained on standard Drosophila corn meal media (Bloomington Stock Center) and maintained at 25°C on a 12-hour light/dark cycle. The following lines were all acquired from the Bloomington Drosophila Stock Center (BDSC) *Piezo*^*KO*^ (BDSC#, *Piezo*-GAL4 (BDSC#, 78335), Df 8977 (BDSC#, 8977), Df 9355 (BDSC#, 9355), and Df 24403 (BDSC#, 24403) *btl-GAL4* (BDSC#, 8807). *Dube3a*^*15b*^ has been previously published as a Drosophila model for AS [21]. UAS-*Dube3a*-FLAG was constructed in the Reiter lab and is available upon request.

### Gut distention assay

Adult female flies were collected post-eclosion and stored at 25°C for 3–5 days. Before testing, flies were transferred to empty vials containing only a damp kimwipe. Flies were starved for 15–18 hours at 25°C prior to feeding. GFP expressing yeast (The ODIN) were cultured at 33°C for 48 hours on G418 (gibco) yeast extract peptone dextrose (YPD) agar. GFP^+^ yeast were scrapped and layered on top of fly food in vials. Starved flies were transferred to newly created GFP^+^ yeast food vials for one hour. After feeding, flies were incapacitated using FlyNap (Carolina Biological Supply). Lower legs were removed and flies were placed with the abdomen facing up for imaging on a Lieca fluorescent dissecting microscope. Images were collected and uploaded into FIJI (imageJ) for analysis. The crop region of the most distended *Piezo*^*KO*^/*Piezo*^*KO*^ fly was traced, generating a normalization region of interest (ROI). The normalization ROI was then applied to all flies in the analysis to quantify distention through fluorescent intensity.

### Western blot analysis

Protein was extracted from 3–5 day old fly heads through mechanical homogenization in RIPA buffer plus Complete Protease Inhibitor Cocktail (Roche). Samples were spun down at 10,000 × g. 20μg of protein were loaded into each lane of 1.5 mm NuPAGE Bis-Tris 4–12% gels (Invitrogen) and transferred to PVDF membrane (Millipore). PVDF membranes were blocked in Intercept Blocking Buffer (Li-Cor Cat# 927-60001) for one hour. Membranes were incubated overnight with the following antibodies diluted in Intercept Blocking Buffer, α-GAPDH (1:5000, Abcam Cat# ab157156), and α-FLAG (1:1000 Cell Signaling, D6W5B). The following secondary antibodies were used, IRDye 680RD Donkey α-Goat (1:5000, Li-Cor Cat# 926-68074), and IRDye 800CW Donkey α-Rabbit (1:5000, Li-Cor Cat# 926-32213). Membranes were imaged on an Odyssey Infrared Imaging System (Li-Cor).

### Digestive system imaging

Three to five day old flies were incapacitated using FlyNap (Carolina Biological Supply) followed by dissection of crop and intestine in phosphate buffered saline (PBS). Digestive systems were dissected and fixed in 4% paraformaldehyde for 1 h, washed three times 5 minutes each in 1XPBS. Digestive systems were blocked and permeabilized in PBT (1X PBS, 0.1% triton-X, 1% BSA) for one hour. Primary antibodies used were α-GFP (1:500, Protein tech Cat# 50430-2-AP) and α-ELAV (1:100, Developmental Studies Hybridoma Bank Cat# 9F8A9). Secondary antibodies used were AlexaFluor 488 goat α-rabbit (1:500, Invitrogen Cat# A11034) and AlexaFluor 594 goat α-mouse (1:500, Invitrogen Cat# A11032). Digestive systems were mounted with ProLong Gold antifade mounting medium with DAPI (Invitrogen Cat#P36941).

Images were acquired using a Zeiss 710 confocal microscope (Zeiss) located in the UTHSC neuroscience imaging core. All imaging settings remained constant between control and experimental conditions. GFP signal was captured using a 488 nm laser, and *elav* signal was captured using a 568 nm laser. Z-stacks were acquired using 1 μm optical sections. All z-stack projections were transformed using maximum intensity projections using ZEN software (Zeiss). Images were acquired with ZEN software (Zeiss).

### Data analysis

All data analysis was performed using Prism 9 (GraphPad). Gut distention analysis was performed using one-way ANOVA with Dunnett’s multiple comparison test. For all statistical tests we set the α to 0.05. All figures were generated using Adobe Illustrator (Adobe).

## Data Availability

All data generated during this study are included in this manuscript, the supplemental material, or can be made available from the corresponding author upon request.
